# Development of a new risk model for predicting cardiovascular events among hemodialysis patients: Population-based hemodialysis patients from the Japan Dialysis Outcome and Practice Patterns Study (J-DOPPS)

**DOI:** 10.1371/journal.pone.0173468

**Published:** 2017-03-08

**Authors:** Yukiko Matsubara, Miho Kimachi, Shingo Fukuma, Yoshihiro Onishi, Shunichi Fukuhara

**Affiliations:** 1 Department of Artificial Organs, Akane-Foundation Omachi Tsuchiya Clinic, and Hiroshima Medical University, Hiroshima, Japan; 2 Department of Healthcare Epidemiology, School of Public Health in the Graduate School of Medicine, Kyoto University, Kyoto, Japan; 3 Institute for Health Outcomes and Process Evaluation Research (iHope International), Kyoto, Japan; 4 Center for Innovative Research for Communities and Clinical Excellence, Fukushima Medical University, Fukushima, Japan; Osaka University Graduate School of Medicine, JAPAN

## Abstract

**Background:**

Cardiovascular (CV) events are the primary cause of death and becoming bedridden among hemodialysis (HD) patients. The Framingham risk score (FRS) is useful for predicting incidence of CV events in the general population, but is considerd to be unsuitable for the prediction of the incidence of CV events in HD patients, given their characteristics due to atypical relationships between conventional risk factors and outcomes. We therefore aimed to develop a new prognostic prediction model for prevention and early detection of CV events among hemodialysis patients.

**Methods:**

We enrolled 3,601 maintenance HD patients based on their data from the Japan Dialysis Outcomes and Practice Patterns Study (J-DOPPS), phases 3 and 4. We longitudinaly assessed the association between several potential candidate predictors and composite CV events in the year after study initiation. Potential candidate predictors included the component factors of FRS and other HD-specific risk factors. We used multivariable logistic regression with backward stepwise selection to develop our new prediction model and generated a calibration plot. Additinially, we performed bootstrapping to assess the internal validity.

**Results:**

We observed 328 composite CV events during 1-year follow-up. The final prediction model contained six variables: age, diabetes status, history of CV events, dialysis time per session, and serum phosphorus and albumin levels. The new model showed significantly better discrimination than the FRS, in both men (c-statistics: 0.76 for new model, 0.64 for FRS) and women (c-statistics: 0.77 for new model, 0.60 for FRS). Additionally, we confirmed the consistency between the observed results and predicted results using the calibration plot. Further, we found similar discrimination and calibration to the derivation model in the bootstrapping cohort.

**Conclusions:**

We developed a new risk model consisting of only six predictors. Our new model predicted CV events more accurately than the FRS.

## Introduction

Mortality risk among hemodialysis population remains substantially higher than in the general population [1], despite improvements in HD treatment. Cardiovascular (CV) events are a major cause of death and becoming bedridden among HD patients. The relative risk of death due to CV events in hemodialysis patients is reported to be 10 to 30 times that in the general population [2,3]. However, a useful prognostic prediction model has not been developed yet, even though the international kidney guidelines [4,5] recommend early detection and prevention of CV events among HD patients.

The Framingham risk score (FRS) is the most commonly used model for predicting 10-year incidence of CV events in the general population [6,7], accounting for age, sex, blood pressure, smoking habit, total cholesterol (TC) or low-density lipid cholesterol (LDL-C), high-density lipid cholesterol (HDL-C), and diabetes mellitus status. The FRS is useful for encouraging lifestyle modification and promotes early prevention in the general population [8,9].

However, several characteristics specific to HD patients have rendered the FRS unsuitable for use in this particular population. For example, the FRS strongly weights symptoms such as hypercholesterolemia. In the general population, hypercholesterolemia is associated with an increased risk for CV events, while hypocholesterolemia tends to lead to increased incidence of CV events in HD patients paradoxically [10,11]. Further, the FRS doesn’t include any HD-specific risk factors, such as mineral metabolism, including calcium and phosphate levels [12–14], anemia [15], and malnutrition [11,16,17], all of which have been identified as risk factors of CV events. The difference in the degree to which each risk factor contributes to CV events between HD and general populations may also hamper model development [18,19]. These discrepancies in characteristics between general population and HD patients render the FRS inappropriate for use in HD patients.

Given the poor evidence of the utility of the FRS in hemodialysis populations, we developed a new risk model for predicting CV events as an alternative to the FRS, which is more appropriate for use in a general population.

## Materials and methods

### Design, setting, and participants

We used the data from Japan Dialysis Outcomes and Practice Patterns Study (J-DOPPS) phase 3 (2005–2008), and phase 4 (2009–2011) to develop the risk equations and prediction model. For our study, eligible participants were patients aged 18 years or older who had been on maintenance HD for at least 90 days. We excluded subjects with missing data for HD vintage. The J-DOPPS collected demographic information, such as laboratory data, drug information, and dialysis conditions, every four months, and information on hospitalization and death at each occurrence. Our present study using J-DOPPS data complied with the Declaration of Helsinki. All participants in J-DOPPS have provided written informed consent before study enrollment. Data collection was performed in a fashion that maintains patient anonymous at the cording center [20]. This study’s conduct was approved by the Ethics Committee of Tokyo Women's Medical University (Approval Numbers 709, 1178, 1278, 1527,1826, and 2143).

### Candidate predictors

We included both FRS risk factors and several HD-specific risk factors identified in previous studies as candidate predictors. The FRS risk factors were age, diabetes mellitus, smoking habit, fifth Joint National Committee blood pressure category, National Cholesterol Education Program TC (or LDL-C), and HDL-C by gender. The HD-specific risk factors included dialysis vintage, time on dialysis per week, vascular access type, Kt/v, BMI and laboratory data (levels of hemoglobin [Hb], calcium, phosphate, intact parathyroid hormone [iPTH], and albumin).

### Main outcome

The primary outcome was the incidence of composite cardiovascular events, including major adverse cardiovascular events, including hospitalization due to unstable or stable angina or non-fatal myocardial infarction, and all-cause death in one year. We also included all-cause death as a composite outcome. While substance causes of death among hemodialysis patients were related to heart disease, such as heart failure and ischemic heart diseases [21], we did not report these deaths due to a lack of inquiry [22].

### Statistical analyses

With regard to baseline characteristics of participants, continuous data with normal distribution were summarized as means (standard deviation [SD]), continuous variables with skewed data as medians (inter-quartile range [IQR]), and categorical data as proportions.

We used multivariable logistic regression with backward stepwise selection to develop the prediction model [23–25]. We first performed multiple imputation for complementation of missing variables, deriving all candidate predictors. Second, we conducted our analysis using multivariable logistic regression for the full model with imputed values and performed backward elimination of the least significant predictor in order. We stopped regression once the p-value for all predictors was less than 0.10 and then completed our new model. Third, we assigned integer scores to selected predictors based on the beta coefficient from the completed model. The discrimination ability of the model was evaluated based on the c-statistic (area under the receiver operating characteristic [ROC] curve) [26–29]. We also compared the c-statistics of the new model to that of the FRS. The consistency between the incidence predicted by the new model and the incidence observed of composite CV events was assessed using a calibration plot. Additionally, we calculated the sum of the risk scores and compared the results with each model to the actually observed incidence of composite CV events, separating the values into quartiles of grades 1 to 4. We conducted bootstrapping with 1000 resamples to assess the internal validation and performed Rubin’s rule on summary estimates to combine all results from multiple imputed datasets. All statistical analyses were performed using STATA 14.0 (version 14.0; Stata Corp, College Station, TX, USA) software, with 2-sided significance set at 0.05. We followed the TRIPOD statement for reporting [30].

## Results

### Study flow diagram

**Fig 1:**
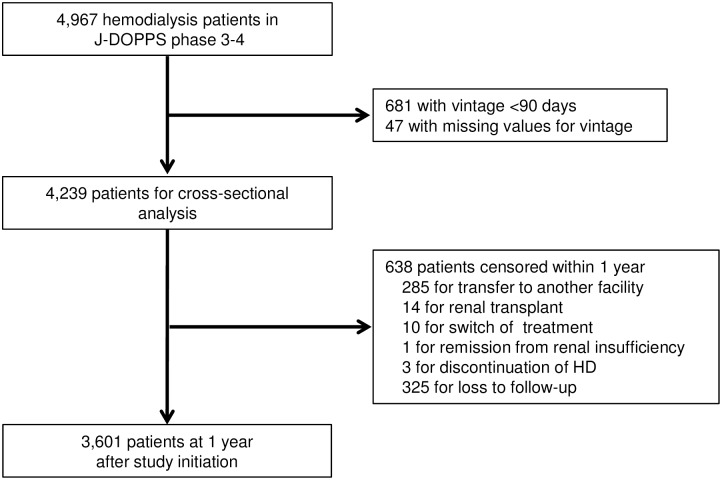
Participant flow diagram and study selection process.

A total of 121 facilities and 4,967 HD patients were included in J-DOPPS phases 3 to 4. Fig 1 shows the participant flow diagram and the process of study selection. After excluding 681 patients for being less than 90 days from initiation of HD and 47 for missing vintage values, 4,239 patients remained in this study at baseline. Within 1 year, 638 patients were censored for reasons other than the primary outcome, such as transfer to another hospital, leaving 3,601 patients ultimately included in the analysis of this study.

### Description of candidate predictors and incidence of composite CV events in the new risk model

The candidate predictors are shown in Table 1. Mean age was 63.7 years, 61.8% of subjects were men, 36.2% had history of CV events, 33.7% had history of diabetes, and the median dialysis vintage was 3.6 years. A total of 328 (9.1%) composite CV events occurred in the year following study initiation (S1 Table). Men had more outcomes (219 of 2,225 [9.8%]) than women (108 of 1,374 [7.9%]).

**Table 1:** Baseline characteristics of patients.

Characteristics	Total (n = 3,601)	Number missing
Age, years	63.7 (12.3)	5
Men, %	61.8	2
Smoker (ever), %	14.2	432
Diabetes, %	33.7	299
History of CV events, %	36.2	268
Pre-dialysis systolic blood pressure, mmHg	150.5 (22.6)	317
Pre-dialysis diastolic blood pressure, mmHg	77.8 (13.6)	322
Dialysis time <720 min/week, %	23.5	484
Vintage, years	3.5 (1.2)	0
Vascular access type, %		748
*AV fistula*	91.7	
* Graft*	7.8	
* Catheter*	0.42	
* *Kt/v	1.4 (0.27)	
Laboratory variables		
*Total cholesterol*, *mg/dL*	155.1 (35.4)	885
* High-density lipid cholesterol*, *mg/dL*	46.8 (17.1)	1,693
* Hemoglobin*, *g/dL*	10.4 (1.2)	309
* Calcium*, *mg/dL*	9.3 (0.83)	488
* Phosphorus*, *mg/dL*	5.5 (1.4)	303
* Intact parathyroid hormone*, *pg/L*	137 (69–236)	1,104
* Albumin*, *g/dL*	3.8 (0.42)	409

Continuous data with normal distribution were summarized as mean (±standard deviation), continuous variables with skewed data were summarized as median (interquartile range), and dichotomous or categorical data were summarized as proportions.

### Derivation of new risk model

We included 17 candidate predictors in our initial model, and the final model ultimately contained only six variables: age, diabetes mellitus status, history of CV events, dialysis time per session, phosphorus level, and albumin level. Beta coefficients of each predictor are shown in Table 2, and scores of selected predictors were assigned an integer score based on the beta coefficient. The smallest beta coefficient that indicated a p value of less than 0.1 in the new risk model was 0.29, which was equivalent to 1 point, and total score ranged from 0 to 20 points.

**Table 2:** Adjusted odds ratios for association between predictors of incidences of composite cardiovascular events (final step of predictor selection).

Characteristics	beta	OR (95% CI)	p value	Score
Age, years				
<55		Reference		0
55–64	0.52	1.7 (1.1 to 2.7)	0.028	2
65–75	0.70	2.0 (1.3 to 3.2)	0.003	2
>75	1.34	3.8 (2.4 to 6.1)	< 0.001	5
Diabetes, %	0.68	2.0 (1.5 to 2.6)	< 0.001	2
History of CV events, %	1.01	2.7 (2.1 to 3.5)	< 0.001	3
Dialysis time <720 min/week, %	0.46	1.6 (1.2 to 2.1)	< 0.001	2
Phosphorus, mg/dL				
<3.5	0.66	1.9 (1.2 to 3.1)	0.008	2
3.5 to <6.0		Reference		0
≥6.0	0.29	1.3 (1.0 to 1.8)	0.045	1
Albumin, g/dL				
<3.0	1.76	5.8 (3.1 to 10.9)	< 0.001	6
3.0 to < 4.0	0.21	1.2 (0.91 to 1.7)	0.18	1
≥4.0		Reference		0

CI, confidence interval; OR, odds ratio

### New model assessment and comparison with the Framingham model

**Fig 2:**
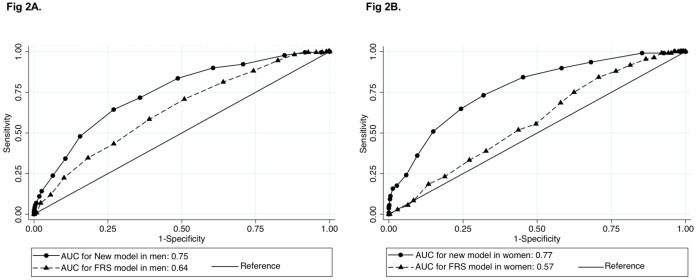
Comparison discrimination ability of new risk model with Framingham model by gender. Results described are c-statistics. (A) The comparison between the new model and Framingham model in men (n = 2,224). (B) The comparison in women (n = 1,372). Circles indicate the AUC of the new model, and triangles indicate that of the FRS model.

We assessed the discrimination ability of the new model using an area under ROC curve(c-statistics: 0.76, 95% CI: 0.73 to 0.78; S1 Appendix) and compared the new model with the Framingham model. ROC curves were compared by sex between our model and the FRS. C-statistics in our new model were significantly higher than in the Framingham model for both men (c-statistics: 0.75, 95% confidence interval [CI]: 0.71–0.78 for new model; 0.64, 95% CI: 0.60–0.68 for FRS model) and women (c-statistics: 0.77, 95% CI: 0.73–0.82 for new model; 0.57, 95% CI: 0.52–0.62 for FRS model; Fig 2).

**Fig 3:**
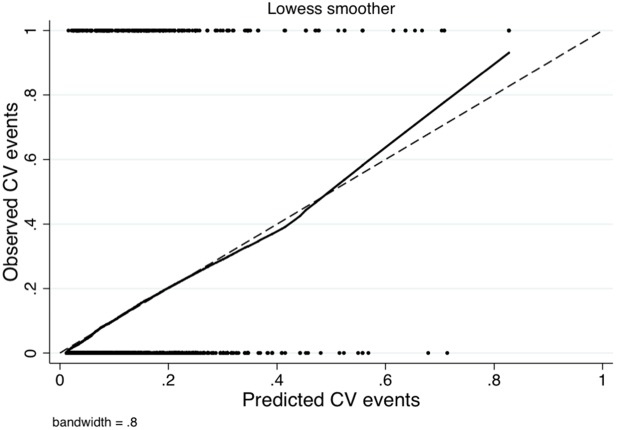
Calibration plot for new model. Result shows the consistency between predicted CV events by new model and observed CV events using a calibration plot. The dotted line indicates perfect fitting, and the solid line indicates the predicted probabilities.

**Fig 4:**
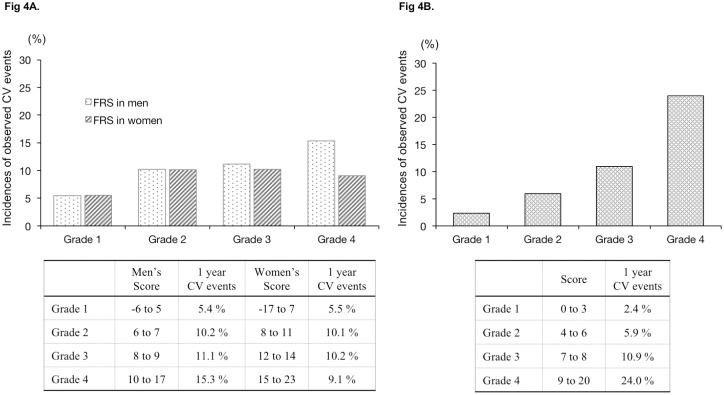
Risk score and incidence of observed CV events in each model. Results shows the association between the risk score and observed CV events. Scores were four groups based on risk score quartile (Grade 1 to 4). Fig 4A shows Framingham risk score by gender. Fig 4B shows new risk score.

Fig 3 shows the accuracy of our model by comparing the incidence predicted by the new model with the actual observed incidence of composite CV events using a calibration plot. Additionally, we divided participants into four groups based on risk score quartile (Grades 1 to 4) obtained from the FRS or the new risk model and compared the observed incidences of composite CV events between the FRS and our new model. The FRS in men ranged from -6 to 17 points (Grade 1: -6 to 5 points, Grade 2: 6 to 7 points, Grade 3: 8 to 9 points, and Grade 4: 10 to 17), while that in women ranged from -17 to 23 points (Grade 1: -17 to 7 points, Grade 2: 8 to 11 points, Grade 3: 12 to 14 points, and Grade 4: 15 to 23); the new risk model score in bothe men and women ranged from 0 to 20 points (Grade 1: 0 to 3 points, Grade 2: 4 to 6 points, Grade 3: 7 to 8 points, and Grade 4: 9 to 20 points). The risk score quartiles obtained from the FRS showed no obvious association with the observed incidence of composite CV events (men: Grade 1: 5.4%, Grade 2: 10.2%, Grade 3: 11.1%, and Grade 4: 15.3%; women: Grade 1: 5.5%, Grade 2: 10.1%, Grade 3: 10.2%, and Grade 4: 9.1%), whereas the quartiles obtained from the new model showed a dose-dependent association with the observed incidence of composite CV events (Grade 1: 2.4%, Grade 2: 5.9%, Grade 3: 10.9%, Grade 4: 24.0%) (see Fig 4).

We assessed the internal validation via the bootstrap method and found that the c-statistics were similar between our new model and the modified new model developed using the bootstrap method (c-statistics: 0.75, 95% CI: 0.74 to 0.76 for bootstrap model), and the calibration plot was also similar (S2 Appendix).

## Discussion

In the present study, we developed a new prognostic prediction model of composite CV events tailored for HD patients including HD-specific predictors such as age, diabetes mellitus status, history of CV events, dialysis time per session and phosphate and albumin levels. Our new model had good discrimination and calibration ability in the derivation cohort, and also showed good discrimination and calibration in the bootstrapping cohort. The score obtained using our new model had a more accurate dose-dependent association with observed CV events than the FRS. We therefore believe that our new model will facilitate earlier detection of incidence risk of composite CV events, potentially improving prognosis.

Previous studies have noted that the FRS cannot adequately predict risk of CV events among HD patients [31], even after incorporating HD-specific risk factors such as ankle brachial index [18], metabolic syndrome status, and albuminuria status [19]. Further, several risk factors included in the FRS are not necessarily risk factors for HD patients at all. For example, while high TC and LDL-C levels are reported to be risk factors for CV events in the general population, low TC and LDL-C levels have been implicated in risk of death due to CV events in HD patients [10,11]. No useful prediction model that comprehensively includes HD-specific risk factors, such as anemia [32–35], malnutrition [16,17,36], and mineral abnormalities [12–14,37] has yet been developed, which have been reported to be strongly correlated with incidence of CV events and mortality in HD patients. Dialysis physicians cannot use FRS because important potential risk factors are not included in the FRS score. More appropriate risk scores are therefore needed to identify hemodialysis patients who experience asymptomatic and untreatable CV events.

Regarding implications for clinical practice, we believe that our new risk model might facilitate more adequate prediction of CV risk, allowing physicians to perform early intervention in asymptomatic HD patients before their condition becomes untreatable. Further, previous studies reported that almost half of HD patients have significant coronary artery stenosis at the time of HD initiation [38–41], a condition which can be exacerbated by dialysis [40]. We therefore believe that more accurate prediction using our new risk model will lead to improved prognosis for a substantial number of HD patients.

Several strengths to the present study warrant mention. First, we developed a novel prognostic prediction model consisting of six exhaustive predictors, including HD-specific risk factors. Our model includes not only age and diabetes mellitus status derived from the FRS, but also history of CV events, dialysis time per session and phosphate and albumin levels as new factors. Second, all of six predictors are commonly measured in most HD facilities, so this new prediction model should be both suitable and easy to use in actual clinical practice. Third, unlike the FRS, our model was developed in a study of patients with history of CV events and those aged over 75 years. Given that a considerable number of HD patients have history or risk of CV events and tend to be older than the general population, this expansion of inclusion criteria is reasonable and improves generalizability [42–44]. Finally, the total number of samples in J-DOPPS was relatively large. All risk factors, including medical history, complications, laboratory data, drug information, and dialysis conditions, were collected at baseline or every four months. Further, the incidence of CV events as a primary outcome was much higher than in previous studies involving participants without renal impairment. We were therefore able to include multiple predictors in our model.

This study also had several limitations. First, the follow-up period for our model (1 year) was much shorter than that of the FRS (12 years). However, we feel this shorter period was justified given that the average life expectancy for HD patients is markedly shorter than that of the general population, with relatively few surviving more than 10 years even with improvements in HD therapy [45]. Second, missing values were quite frequent for several predictors, such as TC and HDL-C, as shown in Table 1, and we performed multiple imputation to construct the prediction model. However, results before and after multiple imputation were consistent, so we believe that the missing values had no marked effects on our findings. Third, the generalizability of our prediction model for populations of other races may be limited. However, while the incidence of CV events among Japanese is low compared to other races [44], we recognized an association between composite CV events and several predictors included in our model. Further, we consider that as for the risk factors included in new model, there is no significant difference among races. Therefore, we believe that our model will still be relevant in countries with a higher prevalence of CV events than Japan. Finally, we have not assessed the external validation of this model yet. We plan to perform this analysis using data from J-DOPPS phases V and VI, which are in progress.

## Conclusions

We developed new model for predicting risk of CV events in HD patients. Our model, which includes HD-specific factors, may be useful for evidence-based management for risk factors of CV events in HD patients. We believe that this prediction model will be more appropriate than the FRS for HD patients, facilitating earlier therapeutic intervention in this population than is possible at present. In the future, we will continue to validate our model in a larger number of subjects.

## Supporting information

S1 Appendix Discrimination ability of new risk model for the incidence of composite CV events in one year after study initiation. (PDF)

S2 Appendix Internal validation using bootstrap method for new model. (PDF)

S1 Table Characteristics of main outcome. (PDF)
