# Supplementary figures and images for: Development of a new risk model for predicting cardiovascular events among hemodialysis patients: Population-based hemodialysis patients from the Japan Dialysis Outcome and Practice Patterns Study (J-DOPPS)
Source: PLoS One. 2017 Mar 8;12(3):e0173468. doi: 10.1371/journal.pone.0173468 (PMC5342257; doi:10.1371/journal.pone.0173468)

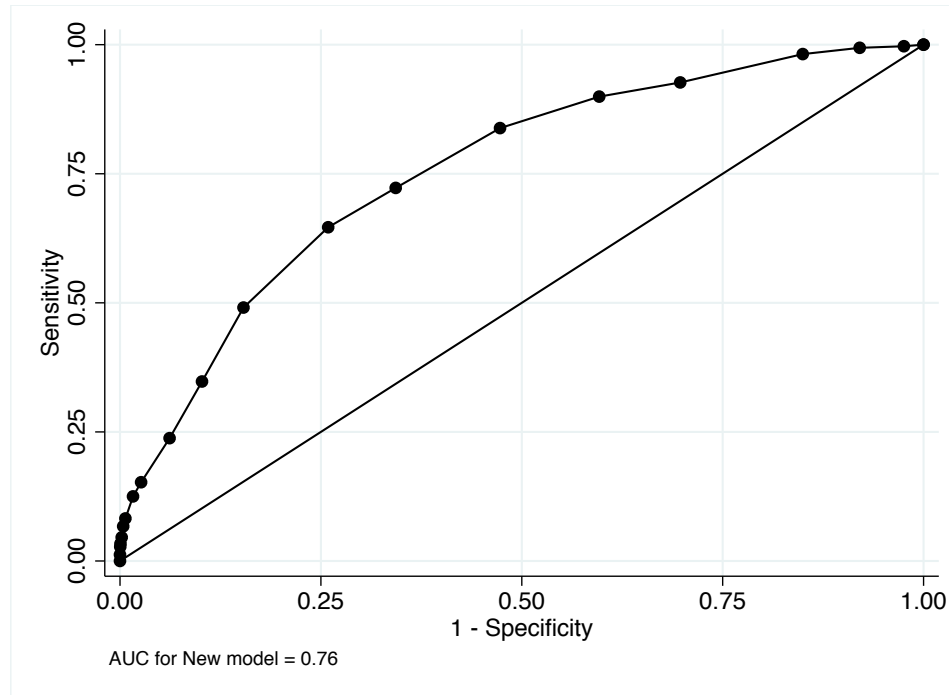

**S1 Appendix.**

Supplement: S1 Appendix — (PDF) [file pone.0173468.s001.pdf]

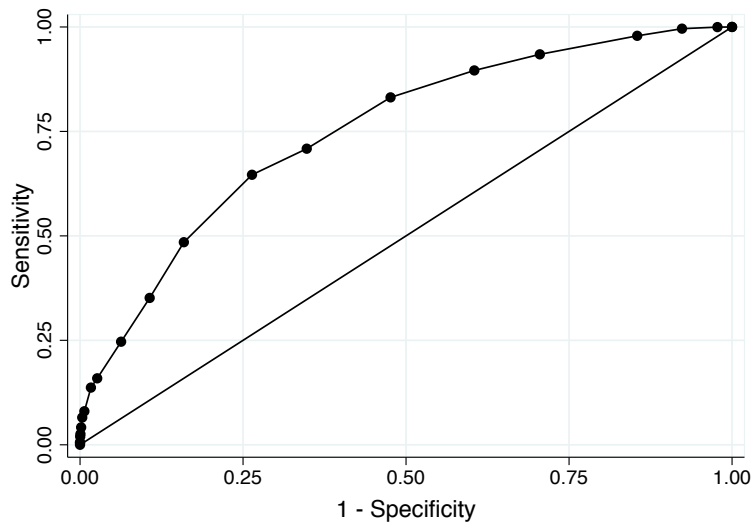

AUC for New model using bootstrap method = 0.75

**S2A.**

**S2 Appendix.**

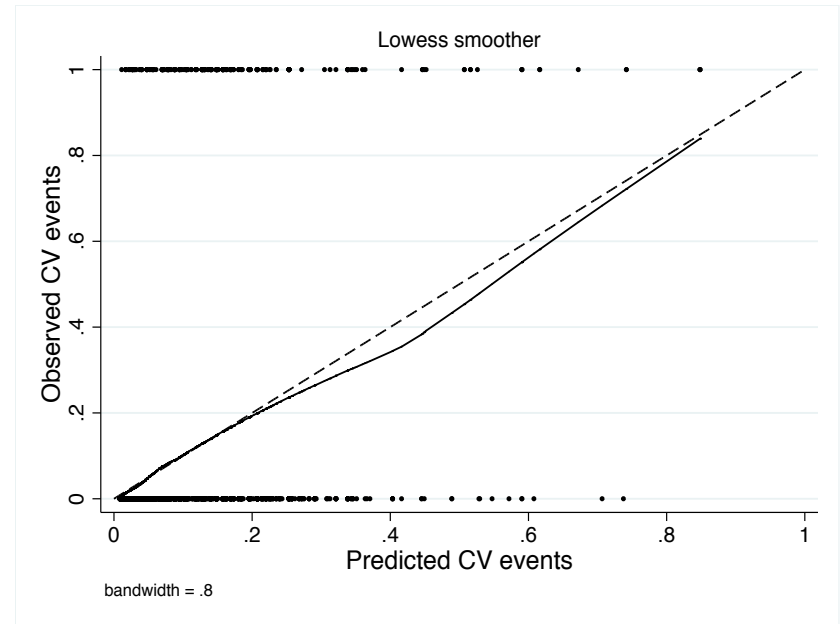

**S2B.**

Supplement: S2 Appendix — (PDF) [file pone.0173468.s002.pdf]
