# Supplementary material for: Development of a new risk model for predicting cardiovascular events among hemodialysis patients: Population-based hemodialysis patients from the Japan Dialysis Outcome and Practice Patterns Study (J-DOPPS)
Source: PLoS One. 2017 Mar 8;12(3):e0173468. doi: 10.1371/journal.pone.0173468 (PMC5342257; doi:10.1371/journal.pone.0173468)
Supplement: S1 Table — (PDF) [file pone.0173468.s003.pdf]

S1 Table. Characteristics of main outcome

Characteristics	Total (n=3,601)
No incidence of mortality, n (%)	2,970 (82.5)
Hospitalization due to CV events, n (%)	231 (5.9)
All-cause death, n (%)	418 (11.6)
CV events	78 (18.7)
Other cardiac events	16 (3.8)
Cerebrovascular diseases	39 (9.3)
Other vascular diseases	11 (2.6)
Infectious diseases	71 (17.0)
Gastrointestinal diseases	7 (1.7)
Liver diseases	5 (1.2)
Cancers	33 (7.9)
Others	37 (8.9)
Unknown	121 (29.0)
